# Reorganisation of rhizosphere soil pore structure by wild plant species in compacted soils

**DOI:** 10.1093/jxb/eraa323

**Published:** 2020-07-15

**Authors:** Jasmine E Burr-Hersey, Karl Ritz, Glyn A Bengough, Sacha J Mooney

**Affiliations:** 1 Division of Agricultural & Environmental Sciences, School of Biosciences, University of Nottingham, Sutton Bonington Campus, Leicestershire, UK; 2 The James Hutton Institute, Invergowrie, Dundee, UK; 3 School of Science and Engineering, University of Dundee, Dundee, UK; 4 CSIRO Agriculture and Food, Australia

**Keywords:** *Cirsium vulgare*, dandelion, *Plantago lanceoloata*, porosity, ribwort plantain, roots, soil compaction, spear thistle, *Taraxacum officianale*, X-ray computed tomography

## Abstract

Soil compaction represents a major impediment to plant growth, yet wild plants are often observed thriving in soil of high bulk density in non-agricultural settings. We analysed the root growth of three non-cultivated species often found growing in compacted soils in the natural environment. Plants of ribwort plantain (*Plantago lanceolata*), dandelion (*Taraxacum officinale*), and spear thistle (*Cirsium vulgare*) were grown for 28 d in a sandy loam soil compacted to 1.8 g cm^–3^ with a penetration resistance of 1.55 MPa. X-Ray computed tomography was used to observe root architecture *in situ* and to visualise changes in rhizosphere porosity (at a resolution of 35 μm) at 14 d and 28 d after sowing. Porosity of the soil was analysed within four incremental zones up to 420 μm from the root surface. In all species, the porosity of the rhizosphere was greatest closest to the root and decreased with distance from the root surface. There were significant differences in rhizosphere porosity between the three species, with *Cirsium* plants exhibiting the greatest structural genesis across all rhizosphere zones. This creation of pore space indicates that plants can self-remediate compacted soil via localised structural reorganisation in the rhizosphere, which has potential functional implications for both plant and soil.

## Teaser

Soil porosity is increased within the rhizosphere of three common wild plant species growing in compacted soil, with significant differences between species.

## Introduction

Pioneer vascular plant species often face particular challenges in colonising new habitats if the topsoil is impoverished with respect to nutrient or water supply, or if the subsoil is compacted and resistant to root penetration and development. Soil compaction is common in agricultural soils, arising as a result of farm traffic (Soane *et al.*, 1981; Hamza and Anderson, 2005) or livestock trampling (Mulholland and Fullen, 1991; Mohseni Saravi *et al.*, 2005), and is acknowledged as a major constraint to crop root growth (Rosolem *et al.*, 2002; Tracy *et al.*, 2011) and productivity (Iijima *et al.*, 1991; Oussible *et al.*, 1992; Chen and Weil, 2011). The phenomenon also occurs in natural ecosystems, for example as a result of trampling by wild animals (Thomas, 1943; Cumming and Cumming, 2003; Lei, 2003; Hosokawa, 2010), even by relatively small mammals (Pascual *et al.* 2017), wallowing (Gutterman, 2003; McMillan *et al.*, 2011; Ozan and Borrero, 2018), or as a result of human activities such as walking and cycling (Lei, 2004). Naturally compact soil horizons can also be formed as a result of glaciation (Fitzpatrick, 1956). Vegetation that develops on such compacted soils is typically distinct from that which prevails in adjacent zones in both natural (Gibson 1989; Trager *et al.* 2004; Alberti *et al.*, 2010) and human-induced contexts (Scott *et al.*, 2005). However, few studies consider the extent to which plants are able to colonise such soils or carry traits that enable them to grow in such dense substrata, such as via aerenchyma formation (Engelaar *et al.*, 1993). Adapting to grow in adverse conditions such as compacted tyre tracks in agricultural fields or roadside verges probably requires specialised root traits. These may be manifest as the ability to radially expand under high levels of mechanical impedance and quickly and effectively occupy a soil volume during establishment (Hettiaratchi, 1990). The ability to outcompete other species due to their adaptive traits enables such plants to grow in suboptimal conditions. For example, it has been shown that deeper root penetration within a soil profile improves the ability of a plant to compete for soil resources in conditions where availability is limited (Place *et al.*, 2008). It has been suggested that roots with greater diameter may be better able to penetrate compacted soils (Materechera *et al.*, 1991; Clark *et al.*, 2003; Chen and Weil, 2009), and hence species with tap roots could be well adapted to grow in such circumstances. In a study investigating root growth of 22 crop species in a highly compacted soil, root extension in all species was severely inhibited, but the roots of dicots seemed to be less restricted than those of monocots (Materechera *et al.*, 1991). Actively growing crop roots have been shown to have the ability to significantly alter the environment in the immediate vicinity of their surface; for instance, they have the potential to ameliorate soil in poor physical condition (Cresswell and Kirkegaard, 1995). To what extent wild species are able to do this is essentially unknown.

X-Ray computed tomography (CT) has been used to investigate the undisturbed rhizosphere, with a focus on root–soil contact (Schmidt *et al.*, 2012) and soil pore formation (Helliwell *et al*., 2017, 2019; Rabbi *et al.*, 2018; Schluter *et al.*, 2018; Koebernick *et al.*, 2019). These studies have examined the impacts crop plants such as wheat, barley, tomato, pea, bean, and chickpea have on soil structure, but to date no study has investigated the formation of microscale pore spaces at the root surface created by wild plant species. It has been suggested that the growth of crops may be more sensitive to levels of soil resistance in the rhizosphere zone compared to non-cultivated plants (Cook *et al.*, 1996). Crop plants have generally been selected for their ability to grow in a loosely structured seedbed and as such they may be poorly adapted to develop in compacted soils. Wild plants that are able to grow on compacted soils may carry intrinsic traits that enable them to do so, but little is known about the *in situ* root architecture of such species.

In this study, we investigated three wild dicot plant species, namely *Plantago lanceolata* (ribwort plantain), *Cirsium vulgare* (spear thistle), and *Taraxacum officinale* (dandelion). All exhibit the same basic root architectural development patterns, having a primary taproot with secondary lateral root development. We hypothesised that the different species would produce varying amounts of root biomass under a high-compaction treatment, and would subsequently have varying effects on the soil porosity over time. We grew each species in highly compacted soil, analysed their root architecture in 3D via X-ray CT, and quantified soil porosity at the bulk and rhizosphere scales.

## Materials and methods

### Soil column preparation and planting

Soil from the Dunnington Heath series, a sandy loam (66.4% sand, 18% silt, and 15.6% clay), was collected from The University of Nottingham experimental farm at Sutton Bonington, Leicestershire, UK (52°50´07″N, 1°15´04.0″W). The soil was air-dried and sieved to <2 mm. Columns (100×51 mm height × diameter) were uniformly packed with soil at a moisture content of 16% to a dry bulk density of ~1.8 g cm^–3^ and a penetrometer resistance reading of ~1.55 MPa (1-mm diameter 30° semi-angle cone with 0.8-mm relieved shaft, driven at 4 mm min^–1^). Columns were packed in two 4-cm layers, and after compacting each layer its surface was lightly scarified to ensure homogenous packing and hydraulic continuity within the columns (Lewis and Sjostrom, 2010). Once packed, the columns were weighed and stored in airtight polythene wraps for 2 d before X-ray CT (µCT) scanning to prevent moisture loss and to allow the columns to equilibrate. Seeds of ribwort plantain (*Plantago lanceolata* L.) (procured from Herbiseed, Reading, UK), dandelion (*Taraxacum officinale* L. Weber ex F.H. Wigg) (collected from the Attenborough Nature Reserve, Nottingham, UK, May 2016), and spear thistle [*Cirsium vulgare* (Savi) Ten.] (collected from Highfields Park Boating Lake, Nottingham, UK, May 2016) were used in the study. For each species, six soil columns were planted with one pre-germinated seed at a depth of ~1 cm, and an additional three columns were left empty as unplanted controls. Of the planted columns, three replicates were scanned and three remained as unscanned planted controls. This allowed the potential effects of X-rays on plant development to be assessed. After planting, all 21 columns were placed in a growth chamber in a randomised block design, with three blocks each including one replicate of each treatments. The growth chamber had a photoperiod of 16/8 h at 270 µmol m^–2^ s^–1^ and day/night temperatures of 20/15 °C, with constant 70% humidity. Plants were grown for 28 d and the subset of 12 columns initially scanned on Day 0 were rescanned on Days 14 and 28. All columns received 4 mg of water each day.

### X-ray computed tomography scanning

Columns were scanned using a Phoenix v|tome|x m X-ray 240 kV (GE Measurement & Control Solutions, Wunstorf, Germany) at the Hounsfield Facility at the University of Nottingham, UK. Voxel resolution was set at 35 μm, potential energy of 160 kV and current of 180 uA, with a 0.1-mm copper filter. Projection image averaging and skip were set to 3 and 1, respectively. Columns were scanned in two sections with a total scan time of 1.25 h. The distance from the sample to the source was 135 mm. For each column, 3000 image projections were captured. Scanned images were optimised to correct for any movement of the sample during the scan and noise was reduced using the beam hardening correction algorithm set at 8. Images were reconstructed at 32 bit using the Phoenix datosx 2.0 reconstruction tool to form a 3D volume, which was visualised as 18-bit data using the VGStudio MAX 2.2.5 software (https://www.volumegraphics.com/).

### Destructive harvest

After 28 d all shoot material was removed and placed in an oven at 50 °C for 48 h to determine dry mass. Roots were washed over a 500-µm sieve and the fresh mass was determined, and then they were stored in 1:1 water:ethanol prior to scanning using a WinRHIZO system (Regent Instruments Inc.). For the scanning, the roots were separated as far as was practically possible in water on A3-size plastic trays and then imaged using an Epson Expression 11000XL flatbed scanner at 800 dpi to determine total root length and mean root diameter. After scanning, roots were dried at 50 °C for 48 h and dry mass was determined.

### 3D image processing, segmentation, and analysis

#### Root analysis

An automated image processing algorithm, RooTrak (Mairhofer *et al.*, 2012), was used to extract roots from the μCT greyscale images. The method employs a tracking-based strategy that follows the root cross-sections through a sequence of images, with the initial digital processing start point being identified manually by the user. As the image stack is traversed, the cross-sections move around the image, reproducing the shape of the scanned root (Mairhofer *et al.*, 2015). The tolerance values were adjusted following visual inspection to ensure that only root material was included in the region of interest (ROI) produced. Once the root system was successfully extracted from the images, it was transferred as a bitmap (.bmp) image stack to VGStudio MAX for cleaning and manual editing. This ensured that the full root system from within each of the columns had been identified. The finalised volumetric representation of the root system architecture was analysed to quantify traits in RooTrak v. 0.3.9.1 (Mairhofer *et al.*, 2012). Global traits derived from the entire root system were root volume, root surface area, maximum root exploration depth (limited by the length of the column), and maximum root exploration width (limited by the width of the column). Additional traits were derived from the individual roots and included the number of lateral roots protruding from the primary root (lateral number) and the length of the taproot, which were subsequently calculated using VGStudio MAX.

#### Soil porosity analysis

Image stacks of each soil column overlaid with a root mask derived from the RooTrak segmentations (Supplementary Fig. S1 at *JXB* online) were exported as TIFFs and imported into ImageJ v. 1.53a (https://imagej.nih.gov/ij/), which was used to manipulate the datasets for subsequent thresholding and analysis using the bin bi-level approach in the open access software Quantim v. 4 (http://www.quantim.ufz.de/) (Vogel and Kretzschmar, 1996). The image stacks were cropped to form new ROIs within the column with dimensions of 1000×1000 pixels by (3.5×3.5 cm). Two thousand images per stack were exported to give the output dataset a representative depth of 7 cm (7 cm out of the 9-cm column length was used as a representative proportion of the soil columns). Once the dimensions of the ROI had been set, the image stack was prepared by: (i) enhancing brightness and contrast, (ii) applying a median filter with a radius of 2 pixels, (iii) converting the image stack to 8-bit data, and (iv) saving the image stack and exporting. The final exported volume for analysis was 3.5×3.5×7 cm, a total of 85.8 cm^3^. Once the image stacks had been prepared, the Li Threshold method in ImageJ was applied to each image sequence to determine two threshold values for each image stack (T_1_ and T_2_; Vogel *et al.*, 2010). This process was repeated for all the scanned columns across the three times points, making a total of 45 sampled columns and 90 individual sections. Quantification of the 3D pore characteristics for whole-column samples was undertaken using Quantim following a similar method to that of Bacq-Labreuil *et al.* (2018). The 3D characteristics (also known as Minkowski functions) were: (i) the percentage of pores with a size greater than the scanning resolution (35 µm), which we refer to as the total soil porosity (for the given resolution); (ii) the pore size distribution as normalised by the total pore volume, expressed as a cumulative value; and (iii) the pore connectivity, determined by the Euler number normalized to the total volume (Vogel *et al.*, 2010): the more negative the Euler number, the greater the pore connectivity.

#### Rhizosphere porosity analysis

**Fig. 1.**
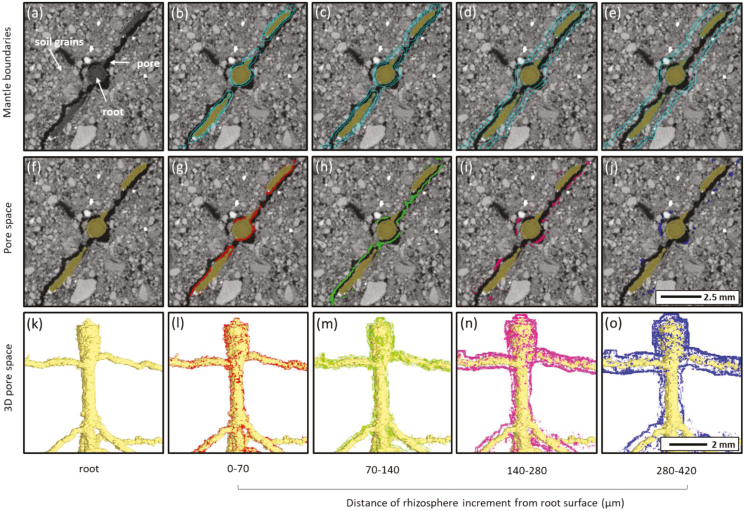
Delineation of rhizosphere zones, and examples of 2D and 3D identification and visualisation of pore spaces for *Cirsium vulgare*. The images were produced using VGStudio MAX v.2.2.5. (a) Original greyscale imagery at Day 28 with root, pore space, and soil indicated. In the other colour images, the root appears as olive green. (b–e) Boundaries of the incremental zones (referred to ‘mantles’), depicted by the blue lines. The zones are indicated at the bottom of the figure. (f–j) Pore spaces in the mantle zones. Different colours correspond to differences in distance from the rhizosphere. (k–o) 3D representations of soil porosity in the mantle zones. Different colours correspond to differences in distance from the rhizosphere.

A method adapted from Helliwell *et al.*, (2017) was used to assess local changes in porosity with distance from the root surface. In VGStudio MAX, the surface determination tool based on greyscale levels was applied to each column to identify air spaces and soil material. Calibrations of the tool were performed by defining material from example areas. Once the pore space had been identified, four new ROIs were created that encompassed incremental zones with distance from the originally segmented root system in 3D (Fig. 1a–e). The segmented root (ROI) was dilated by +2, +4, +8, and +12 pixels (equivalent to 70, 140, 280, and 420 µm distance from the root). This resulted in four incremental section volumes that were 0–70, 70–140, 140–280, and 280–420 µm distal from the root surface, which we term ‘mantles’, for which the total pore volume was identified (Fig. 1f–j). The porosity of each mantle was expressed as pore volume as a percentage of the total incremental mantle volume. These pore volumes were then false-coloured for visualisation of the pore locations within each mantle layer (Fig. 1k–o). This process was undertaken on each of the nine scanned plants at two time-points, resulting in 72 individual rhizosphere porosity mantles for analysis. All volume manipulation and analyses were performed in VGStudio MAX.

### Statistical analysis

One-way factorial ANOVA was conducted on the 2D WinRHIZO data. Two-way ANOVA with specified block design was conducted on the X-ray CT scanned plants to assess whether time or species had the greatest effect on the root characteristics of the plants. Regression analysis was performed on the X-ray CT and WinRHIZO root data. To assess the soil characteristics including porosity, pore connectivity, and pore-size distribution, two-way ANOVA was performed with a treatment structure of time × pore diameter classification with a specified nested block structure. To assess the porosity of the rhizosphere mantles, two-way ANOVA was performed with a treatment structure of species × time with a specified nested block structure.

## Results

### Root characteristics

**Fig. 2.**
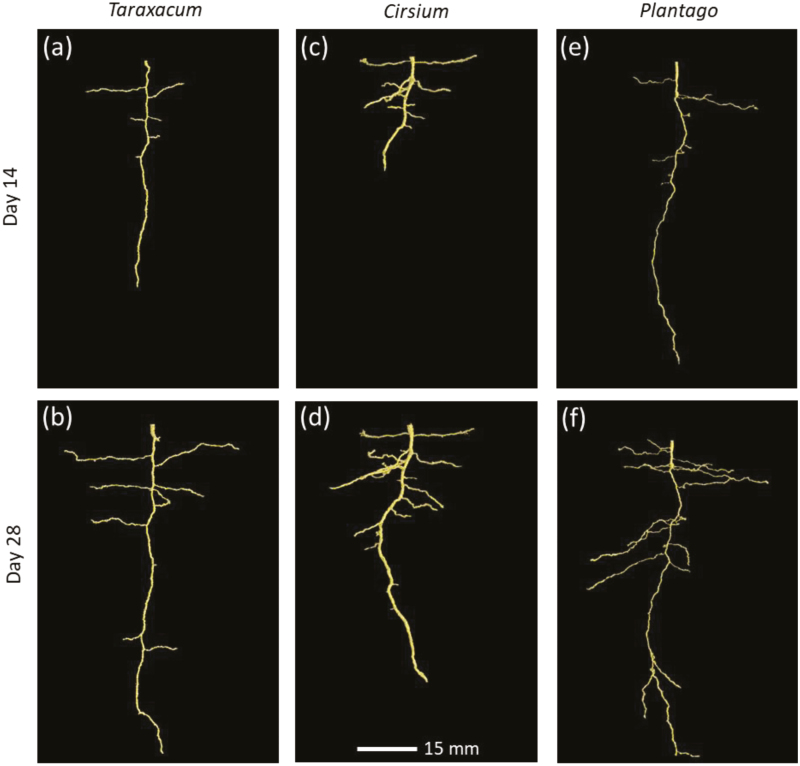
Representative 3D renderings using VGStudio Max V.2.2.5 of root systems grown in repacked soil columns with a bulk density 1.8 g cm^–3^. Plants of *Taraxacum officinale*, *Cirsium vulgare*, and *Plantago lanceolata* are shown at 14 d and 28 d growth, as indicated. (This figure is available in colour at *JXB* online.)

**Fig. 3.**
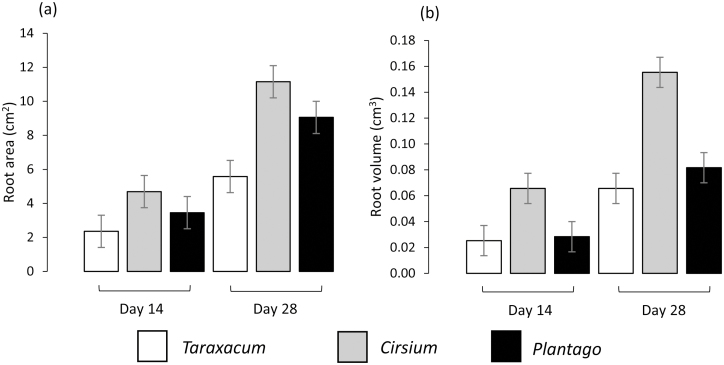
(a) Mean surface area and (b) volume of roots of *Taraxacum officinale*, *Cirsium vulgare*, and *Plantago lanceolata* grown for 14 d and 28 d in repacked soil columns with a bulk density 1.8 g cm^–3^. The data are derived from X-ray computed tomography segmentation and are means (±SE) of 6 replicates.

3D visualisations of the root systems of the three species were analysed based on renderings of the X-ray CT data on Days 14 and 28. There was a marked similarity in the root architecture of the species, with all producing vertical primary taproots with subsequent lateral root formation. On Day 14 there was a distinguishable morphological difference between *Cirsium* and the other two species, with its root system exploring a smaller volume of the soil column and producing more lateral roots than *Taraxacum* or *Plantago* (Fig. 2a, c, e). By Day 2, *Cirsium* roots had extended deeper into the soil column and produced a great number of thicker laterals, whilst the roots of *Taraxacum* and *Plantago* were notably finer in comparison (Fig. 2b, d, f). The total area of roots for all species increased significantly over time (*P*<0.001; Fig. 3A). *Cirsium* had the greatest root area on both Day 14 and 28, *Taraxacum* had approximately half the root area compared to *Cirsium* by Day 28, and *Plantago* was intermediate (overall species effect: *P*=0.004; Fig. 3A). The root volume of *Cirsium* was also greater than the other species on both Days 14 and 28 (*P*<0.001; Fig. 3B). Within each time-point, there was no significant difference in root volume between *Taraxacum* and *Plantago*. The maximum depth of the root system increased significantly over time for all species (*P*=0.006), with most the roots of most plants reaching ~8 cm by Day 28. There was no significant difference in the length of the primary taproot between the species at either time-point (mean±SE: 6.35±1.39 cm and 10.3±5.80 cm for Days 14 and 28, respectively).

The maximum exploration width of the root systems significantly increased over time (*P*=0.006) and differed among the species. On Day 14, *Cirsium* had a significantly greater exploration width (mean 4.9 cm) in comparison to *Taraxacum* and *Plantago* (3.4 cm and 4.4 cm, respectively: pooled SE=0.37; *P*=0.007). *Taraxacum* produced notably fewer lateral roots in total (mean 9) in comparison to *Plantago* and *Cirsium* (18 and 19, respectively; SE=19.4; *P*=0.023).

### Biomass

The scanning process using X-ray CT did not affect the dry weight of the shoots or roots (*P*=0.341, *P*=0.991, respectively). *Cirsium* (7.5 mg) and *Plantago* (5.5 mg) produced significantly greater root biomass than *Taraxacum* (2.5 mg) (pooled SE=0.8; *P*<0.001).

### WinRHIZO parameters and visual assessment

Total root length was not significantly different between the species (overall mean 58 cm: pooled SE=13.35; *P*=0.110). Exposure to X-rays reduced the total root length by 35% irrespective of species (*P*=0.020), but did not affect the mean root diameter. *Cirsium* (mean 0.29 mm) produced thicker roots than both *Plantago* (0.27 mm) and *Taraxacum* (0.26 mm: pooled SE=0.013; *P*=0.080). Comparison between the two root identification methods, WinRHIZO and X-ray CT, by regression analysis resulted in an *R*^2^ value of 0.35. Visual assessments indicated that *Cirsium* roots had a substantial rhizosheath that was well adhered to the root system when they were removed from the soil columns. In contrast, *Taraxacum* and *Plantago* had comparatively few soil aggregates attached to their roots.

### Whole-column and rhizosphere pore characteristics

**Fig. 4.**
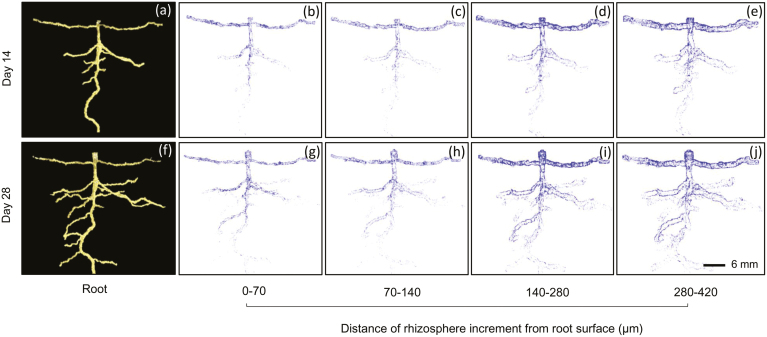
Example renderings of 3D segmented pore space within incremental rhizosphere zones at varying distances from the root surface for *Cirsium vulgare* grown in repacked soil columns with a bulk density 1.8 g cm^–3^. The visualisations were produced using VGStudio MAX v. 2.2.5. (a, f) Example segmented root systems on Days 14 and 28. Pore spaces for (b–e) Day 14 and (g–j) Day 28. (This figure is available in colour at *JXB* online.)

**Fig. 5.**
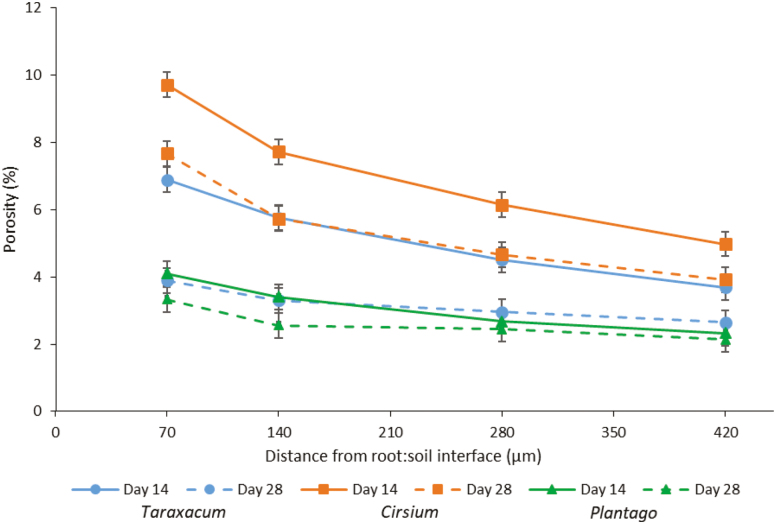
Variation over time in soil porosity within incremental rhizosphere zones at varying distances from the root surface for plants of *Taraxacum officinale*, *Cirsium vulgare*, and *Plantago lanceolata* grown in repacked soil columns with a bulk density 1.8 g cm^–3^. Data are means (±SE) of 6 replicates. (This figure is available in colour at *JXB* online.)

The total soil porosity of the columns (based on a 35-µm resolution) decreased over time, from 1.3 % on Day 0 to 0.7 % on Day 28 (pooled SE=0.19; *P*<0.001). None of the Minkowski functions relating to pore size distribution or pore connectivity were significantly different between the species or time-points (*P*>0.05 in all cases; Supplementary Fig S2). Visualisation of the porosity associated with the rhizosphere showed that it was generally greater around the roots closer to the soil surface, i.e. the older roots, on both Days 14 and 28 (Fig. 4). For all species, the porosity within the incremental rhizosphere mantles was greatest immediately next to the roots and it decreased with distance from the root surface (*P*<0.001; Fig. 5). *Cirsium* exhibited consistently greater rhizosphere porosities on both Days 14 and 28 compared to the other two species, and on Day 14 *Taraxacum* exhibited greater porosity than *Plantago* (*P*<0.001). For all species, the incremental rhizosphere mantle zones had a greater porosity on Day 14 than on Day 28 (*P*<0.001), and this was particularly evident for *Cirsium* and *Taraxacum*. For *Taraxacum,* the biggest decline in rhizosphere pore space occurred in the first mantle, 0–70 µm from the root surface, with the mean porosity decreasing from 6.9 % on Day 14 to 3.9 % on Day 28. For *Cirsium*, the pore space decreased by 2% from the 0–70 µm zone to the 70–140 µm zone. *Plantago* consistently had the lowest observed rhizosphere porosity values, which decreased by less than 1% in each mantle zone from Day 14 to Day 28. Interestingly, the porosity of the furthest mantle from the root surface measured at 350–420 µm for *Cirsium* was greater than that of the first mantle measured at 0–70 µm for *Plantago*.

## Discussion

The volume of soil that can be explored by a root system is directly determined by its architecture (Morris *et al.*, 2017). In principle, certain complex architectures may be the most efficient in acquiring soil-based resources, but the force required to penetrate compacted soil is often constraining (Bizet *et al.*, 2016), restricting the 3D topology of the root system (Tracy *et al.*, 2012; Burr-Hersey *et al.*, 2017). In our study the responses of individual species to compaction was varied, despite there being no overall difference in taproot length. It has previously been observed in barley that when the main axis is impeded, compensatory growth of lateral roots can occur (Goss, 1977). The length and shape of the primary root axis of a species indicates how readily it has been able to displace the soil: the straighter the taproot, the less it has buckled and deflected as it has grown downward. An increase in root diameter, such as we observed in *Cirsium*, might indicate a response to the mechanical impedance of the soil, which results in slower root elongation rates (Bengough and MacKenzie, 1994). *Cirsium* produced a shorter taproot in the first instance and notably more lateral roots compared to the other two species (Fig. 2). The differences in the way in which the species produced different types of root reinforces the utility of the visual assessment of the systems. For example, it was clear that *Cirsium* developed a shallower, thicker root system in comparison to the deeper, finer systems exhibited by *Taraxacum* and *Plantago*.

Traits that enable roots to advantageously modify their surroundings when conditions are sub-optimal are important, especially in relation to colonising physically dense subsoils. For example, thick roots are able to penetrate compacted soil because they buckle less under mechanical stress (Clark *et al.*, 2003). Our study featured an experimentally compacted sandy soil system, with few large pores and no previously extant biopores. As a result, the packed columns presented a dense, granular environment that the roots had to physically displace in order to grow. As soil bulk density increases, the connectivity of the pore system usually decreases (Udawatta *et al.*, 2016), resulting in increased mechanical impedance to the root system (Bengough, 2012). The extent to which a root system can modify the structure of the soil around it will influence the mechanical impedance to root growth and proliferation. Here, we observed a significant increase in porosity but only directly adjacent to the plant roots (Fig. 5), often at the site of lateral-root emergence (Fig. 4), as previously shown by Helliwell *et al.* (2017). This trend was observed across all species but it differed substantially between them, with the greatest increases in rhizosphere porosity being manifest by *Cirsium*. An increase in porosity adjacent to the root surface could be due to small fluctuations in soil matric potential that cause micro-aggregation (Feeney *et al.*, 2006; Albalasmeh and Ghezzehei, 2014), to root-shrinkage during evapotranspiration (Huck *et al.*, 1970; Carminati *et al.*, 2013), or, near the soil surface, to fluctuating lateral mechanical stresses transmitted from the shoot via the stem and taproot. The porosity that we observed decreased with time for all the species, which we attribute to the continued radial expansion of the taproot systems into the newly created pore space. Our results suggest that the species have similar fundamental mechanisms of structural reorganisation at the root surface, but with greater potential for the phenomenon in the case of *Cirsium*. Our experiment was designed to determine whether the selected plants had the ability to grow in compacted soils and, if so, how they modified their local environment. We averaged the rhizosphere porosity data across the entire root systems, and therefore we consider the pore space as being a function of distance from the root surface and not as a function of depth within the column, which would be concomitantly associated with the age of the roots.

Although changes in porosity were not observed at the whole-column scale, they were apparent at the rhizosphere scale, where they would be of significance to the plants. Soil porosity in this zone was inherently greater than that of the bulk soil up to 2.8 mm distance from the root surface (Fig. 5). Rabbi *et al.* (2018) observed a zone of hydraulic influence of approximately 3 mm for roots of chickpea growing in an oxic soil, whilst Helliwell *et al.* (2019) found that the rhizosphere zone of structural influence is variable and is related to the species (i.e. contrasting root systems), soil type (loamy sand versus clay loam), and the level of soil compaction. Kirby and Bengough (2002), Bengough *et al.* (2006) and Chen and Weil (2009) observed that radial expansion of a root helps to relieve stress at the apex, thus decreasing the likelihood of the root buckling. In our study, *Cirsium* developed notably thicker roots than *Taraxacum* or *Plantago*, indicating that it possesses a trait that would potentially enable it to derive greater benefit from this effect. *Cirsium* was also the species that produced the greatest proportion of modified pore space in the rhizosphere (Fig. 5). An increase in the localised pore space adjacent to the roots will modify the liquid and gas phases of the soil system with which the roots are in contact, and may modify transport to the root surface depending on the soil matric potential. Formation of larger pores directly adjacent to the taproot at the soil surface (e.g. Fig. 1), may facilitate the movement of stem-flow rainwater to deeper layers after intense rainfall events. When the soil is relatively dry such that air-filled pore-space forms a hydraulic barrier between the mature taproot and the soil matrix, plants will rely on water uptake from the soil around the younger root tissues nearer the root tip, which are likely to be in closer contact with the soil.

Oleghe *et al.* (2017) showed that increases in the concentration of root exudates can result in decreases in soil penetration resistance. The presence of mucigel, root hairs, and fungal hyphae can be responsible for the agglutination of soil particles to roots (Moreno-Espíndola *et al*., 2007), and it would be of interest to examine these factors in the three species that we studied. A study by Bao *et al.* (2014) suggested that porous rhizosheaths may hold significantly more water at the root–soil contact zone. The overall greater root diameter and rhizosphere pore space that we found (Figs 2, 5) indicated that the presence of the rhizosheath may have positively impacted lateral root emergence (Fig. 4). During this phase of rhizosphere development many root–soil interaction processes occur, one of the by-products of which is the structural reorganisation of soil particles, and this clearly has implications in the context of increasing pore space. Our study indicated that root-induced structural changes in the high bulk-density soil appeared to be confined to the rhizosphere, at least during the first 28 d of growth. The development of pore space along lateral roots (Fig. 1) might subsequently contribute to more substantial generation of soil structure.

Our study involved the use of plant species not previously examined using X-ray CT. Blaser *et al.* (2018) investigated the effects of cumulative X-ray dosage on *Vica faba* and *Hordeum vulgare* over a 17-d period and observed significant decreases in total root length in the former but not in the latter. This highlights that susceptibility to X-ray dosage varies between species, and that for investigations involving temporal resolution an unscanned treatment is required for baseline comparison of root parameters observed in 2D. The only X-ray-induced effect that we observed was a reduction in overall total root length in all the species that we examined in comparison to measurements made using WinRHIZO. Since the effect was consistent across all the species, we surmised that this reduction did not interfere with the determination of relative differences between the species.

## Conclusions

We have demonstrated the ability of selected wild plant species to grow in extremely compacted soils, and shown that they are to create and modify soil pore space, which is likely to be to their advantage as this will provide increased access to both water and air during the early stages of growth. However, this effect was confined to the rhizosphere, i.e. at a scale of µm as opposed to cm. This has implications for the development of successional series where compacted soils are involved, and our results provide information about advantageous root traits that enable colonisation of such habitats. Our results are also of relevance in the context of the potential for using plants to remediate compacted soils in agricultural contexts. Our study was confined to the first 4 weeks of plant growth, and hence further investigations into changes in the rhizosphere zone over extended periods are warranted. The precise mechanisms and associated traits in species such as *Cirsium* that are better able to penetrate extremely compacted soils are also worthy of further study.

## Supplementary data

Supplementary data are available at *JXB* online.

Fig. S1. Example of image masking with *Cirsium* roots at Day 14 for calculating soil porosity.

Fig. S2. Minkowski functions for sandy loam soil at 1.8 g cm^–3^ under the influence of three different plant root systems.

eraa323_suppl_Supplementary_Figures
