# Supplementary material for: Reorganisation of rhizosphere soil pore structure by wild plant species in compacted soils
Source: J Exp Bot. 2020 Jul 15;71(19):6107–15. doi: 10.1093/jxb/eraa323 (PMC7541912; doi:10.1093/jxb/eraa323)
Supplement: eraa323_suppl_Supplementary_Figures [file eraa323_suppl_supplementary_figures.pdf]

Supplementary data

Fig S1. Example of Root Mask for *Cirsium* at Day 14 with overlaying greyscale soil imagery for soil porosity calculation. (a) Original grey scale image, (b) Segmented root system highlighted by blue outline, (c) White root mask overlaying original greyscale image. Images generated using Visual Graphics StudioMAX V2.2.3

Fig S2. Minkowski functions for sandy loam soil at 1.8 g cm $^{-3}$ under the influence of three different plant root systems. (a) Soil porosity (%), (b) Pore size distribution, represented as the % of pores in each pore diameter class within the total pore network, (c) Connectivity of the pore system described by an Euler number. Points represent means, whiskers pooled SE.

Supplementary figures

S1.

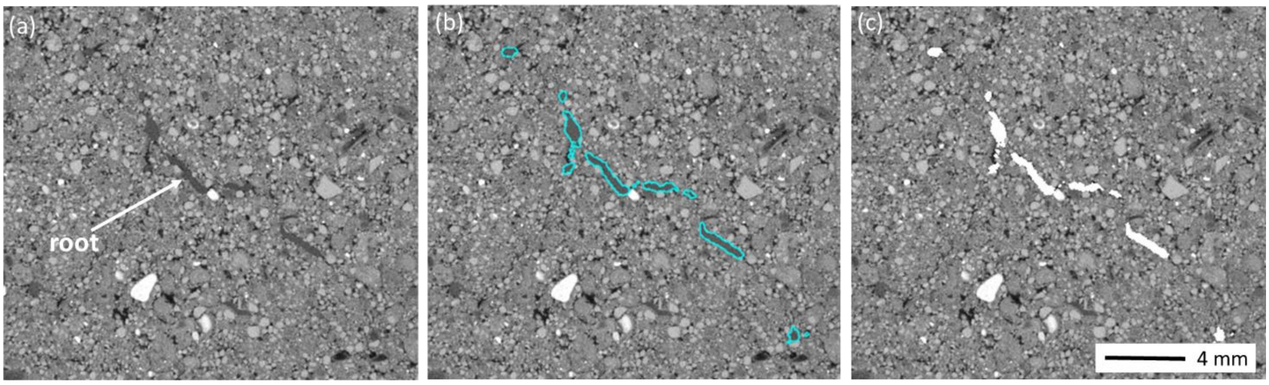

S2.

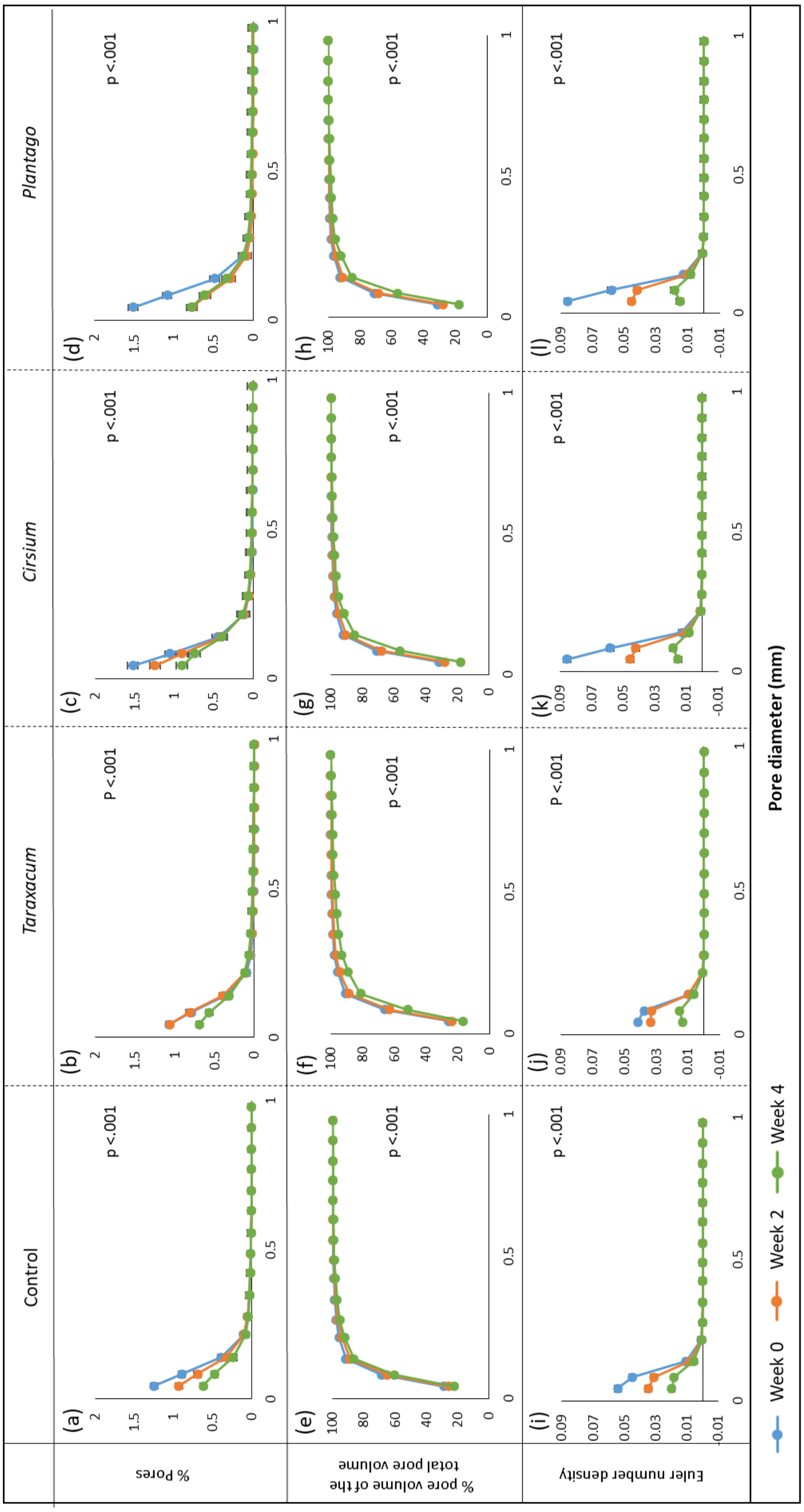

Control
Taraxacum
Cirsium
Plantago
% Pores
% pore volume of the total pore volume
Euler number density
(a)
(b)
(c)
(d)
(e)
(f)
(g)
(h)
(i)
(j)
(k)
(l)
p <.001
Pore diameter (mm)
Week 0
Week 2
Week 4
